# Attack phenotypes and disease course in pediatric MOGAD

**DOI:** 10.1002/acn3.51759

**Published:** 2023-03-31

**Authors:** Jonathan D. Santoro, Timothy Beukelman, Cheryl Hemingway, Suvi R. K. Hokkanen, Frank Tennigkeit, Tanuja Chitnis

**Affiliations:** ^1^ Division of Neuroimmunology, Department of Pediatrics Children's Hospital Los Angeles Los Angeles California USA; ^2^ Department of Neurology Keck School of Medicine at the University of Southern California Los Angeles California USA; ^3^ Epi Excellence LLC Garnet Valley Pennsylvania USA; ^4^ Great Ormond Street Hospital for Children London UK; ^5^ UCB Pharma Brussels Belgium; ^6^ UCB Biosciences Monheim Germany; ^7^ Department of Neurology Mass General Brigham Boston Massachusetts USA

## Abstract

Myelin oligodendrocyte glycoprotein antibody‐associated disease (MOGAD) is an autoimmune demyelinating condition that affects children differently than adults. We performed a literature review to assess the presentation and clinical course of pediatric MOGAD. The most common initial phenotype is acute disseminated encephalomyelitis, especially among children younger than five years, followed by optic neuritis (ON) and/or transverse myelitis. Approximately one‐quarter of children with MOGAD have at least one relapse that typically occurs within three years of disease onset and often includes ON, even if ON was not present at onset. Clinical risk factors for a relapsing course have not been elucidated.

## Introduction

Myelin oligodendrocyte glycoprotein antibody‐associated disease (MOGAD) is an autoimmune demyelinating condition associated with antibodies against MOG (MOG‐Ab) that affects both children and adults. The most common manifestations of MOGAD are acute optic neuritis (ON) with or without transverse myelitis (TM) and, in younger children, acute disseminated encephalomyelitis (ADEM). There can be significant phenotypic overlap with other demyelinating conditions such as multiple sclerosis (MS) and neuromyelitis optica spectrum disorders (NMOSD), making diagnosis and management challenging.^1^, ^2^ Many patients experience a monophasic disease course, while others experience relapses that may differ from the phenotypic manifestations at the first event. It appears that acute attacks of MOGAD and relapsing MOGAD can often be effectively treated with short courses of immunosuppression and chronic immunosuppressive and immunomodulatory therapies, respectively, but no adequately controlled therapeutic studies have been published to date.^3^, ^4^

MOGAD can occur at any age, with important differences in pediatric versus adult onset. Unlike MS, the incidence of MOGAD is higher in children compared with adults.^5^ Multiple studies have also identified that pediatric patients, especially those younger than five years old, more frequently present clinically with ADEM compared with older populations.^6^, ^7^, ^8^ Children have a lower risk for a relapsing course compared with adults, yielding clinical questions regarding the necessity of long‐term therapeutic intervention and disease monitoring.^6^, ^9^ Since clinical testing for MOG‐Ab has been expanded only over the last half decade, greater understanding of MOGAD and its clinical course in children is needed.

Current knowledge of MOGAD's demographic features, clinical phenotypes, and relapse risk is limited, even more so in children, where low patient volumes and lack of multi‐center design limit existing datasets. The authors conducted an extensive review of the published literature of pediatric MOGAD to summarize the current understanding and identify knowledge gaps.

## Methods

### Study design and inclusion criteria

A systematic search of Medline via PubMed and Embase was conducted in October 2020 to identify all articles on pediatric MOGAD. Initial search terms were “myelin oligodendrocyte glycoprotein” or “MOG” in order to optimize a broad article capture. Additional articles were identified by searching bibliographies of included articles and review articles with substantial coverage of pediatric MOGAD. A second comprehensive search of Medline via PubMed was performed in January 2022 to identify any relevant articles published since October 2020. All screening of articles and data extraction was confirmed by a second, independent reviewer.

### Inclusion criteria

Inclusion criteria for abstract level review included: (1) published in English since 2010; (2) human study about MOGAD; (3) full‐length research article (i.e., not scientific meeting abstract, case report, systematic review, narrative review, editorial, commentary, or letter to the editor); and (4) presentation of pediatric‐specific data on five or more patients aged <18 years old. Articles were not excluded for report of overlapping patients from other manuscripts in order to be maximally inclusive and because it was uncertain which specific patients were overlapping. Although diagnostic criteria have been proposed,^10^ there is no standard for routine diagnosis of MOGAD except for detection of MOG‐Ab positivity in a patient with compatible findings; thus, use of specific MOGAD diagnosis criteria was not evaluated. Manuscripts meeting full‐text level review were subsequently reviewed and data were extracted.

### Data extraction

Data from identified articles were extracted using a standard electronic form. Each article was independently and qualitatively assessed for bias in patient selection. Articles with more than 30 pediatric patients were considered “large studies” and were preferentially presented in the results. Data related to patient demographics, initial and subsequent clinical phenotypes, disease severity measures at presentation and follow‐up, and the characteristics of disease relapse including likelihood, frequency, and predictive factors were extracted.

### Definition of severity

No specific disease severity or disability metric exists to assess individuals with MOGAD of any age. For this reason, severity was assessed by report of the expanded disability severity score (EDSS) or the modified Rankin score (mRS), when available. For patients with ON, visual acuity (Snellen and Sloan Letter charts) and the visual function scale of the EDSS were used when available.

### Limitation of selection bias

We attempted to address patient selection bias in the presentation of results. Assessment of initial MOGAD clinical phenotype was restricted to large inception cohort studies. Assessment of MOGAD clinical phenotypes over the disease course was restricted to large inception cohorts with reports of individual patient's subsequent specific disease course. In addition to these restrictive approaches, all studies that reported MOGAD initial and subsequent phenotypes were summarized separately. Only studies that systematically tested for MOG‐Ab at the first demyelinating episode were included in the assessment of the likelihood of relapse. Studies restricted to specific MOGAD phenotypes (e.g., ON) were excluded from the assessment of MOGAD relapse characteristics. Regarding MOGAD relapse characteristics, assessment of number of relapses per patient was restricted to reported means, medians, and distributions (i.e., simple ranges were not considered), and the time to first relapse was assessed using reported medians.

## Results

**Figure 1:**
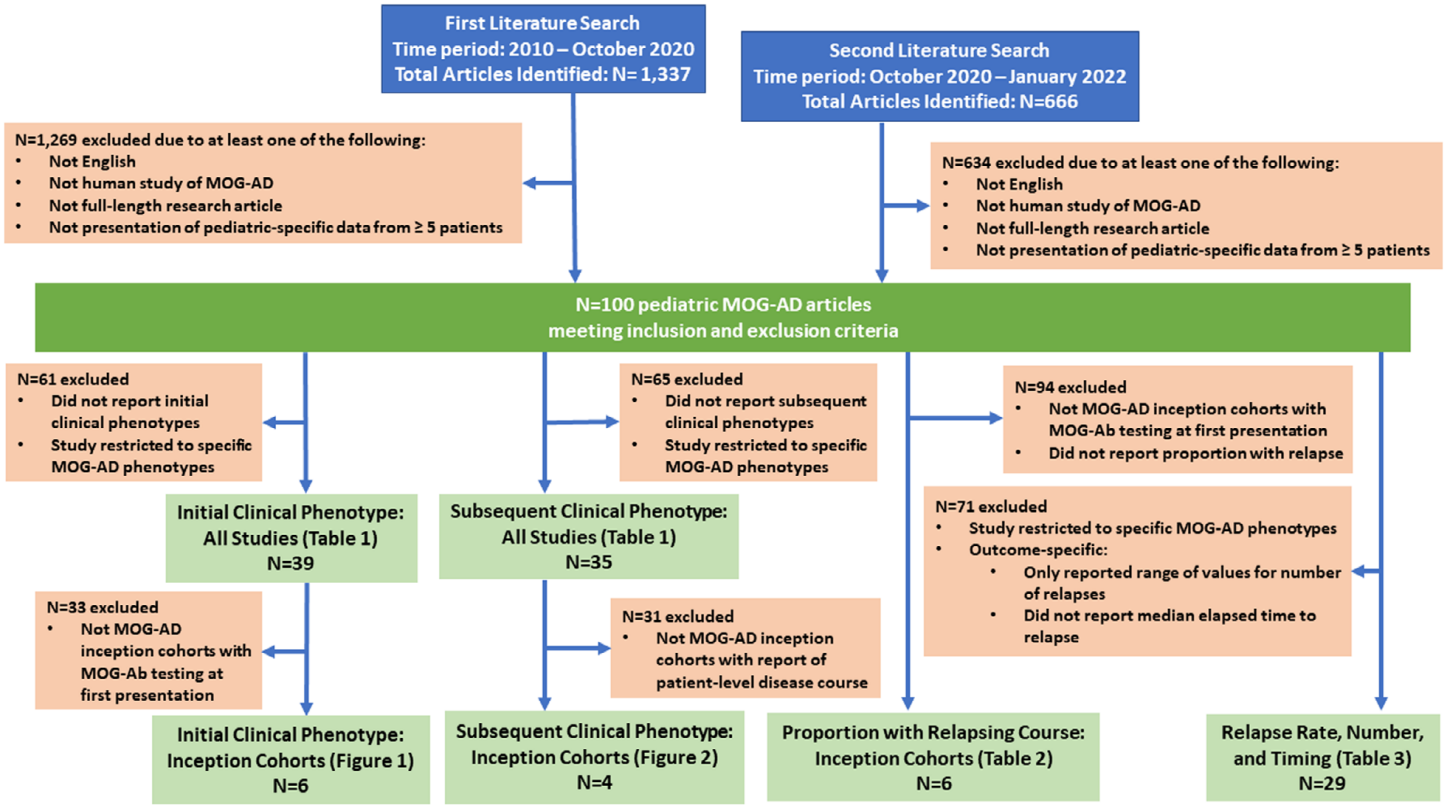
Flow diagram of article inclusion.

In total, 2,003 articles were identified (1,337 in the first search and 666 in the second search). There were 100 articles about pediatric MOGAD that met the inclusion and exclusion criteria. These articles were assessed for inclusion in each of the topics of interest for this report (Fig. 1).

The presence of selection bias was an important consideration in interpreting the results of included articles. Approximately 60% of studies (59/100) attempted to systematically include patients rather than rely upon a convenience sample. Approximately one‐half of studies (48/100) were restricted to patients with specific MOGAD phenotypes, disease course, or clinical management.

### Initial clinical phenotype

**Figure 2:**
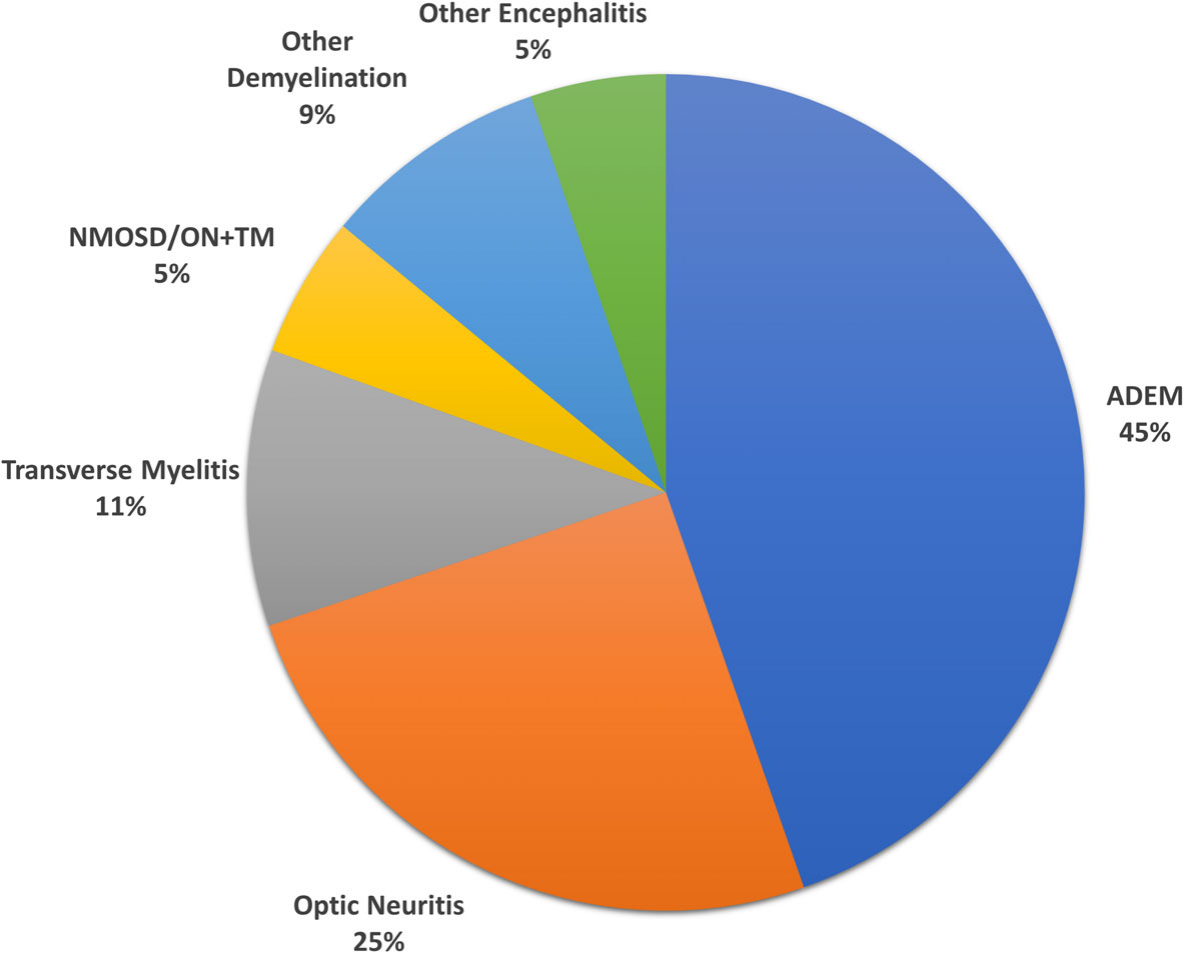
Initial clinical phenotype of first attack in pediatric MOGAD according to large inception cohorts for all pediatric ages (based upon^11^, ^12^, ^13^, ^14^, ^15^, ^16^).

**Figure 3:**
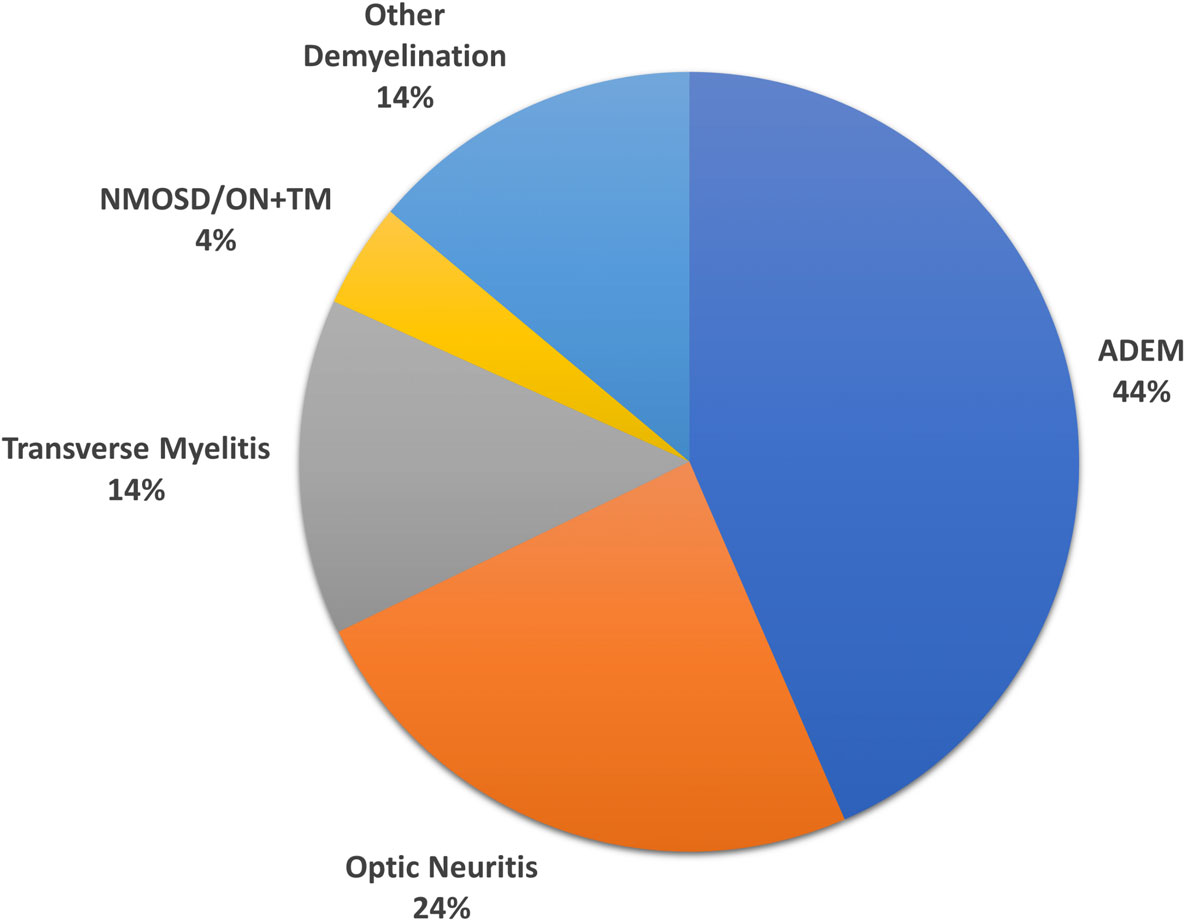
Initial clinical phenotype of first attack in pediatric MOGAD according to large inception cohorts for patients age 0–10 years (based upon^12^, ^15^).

**Figure 4:**
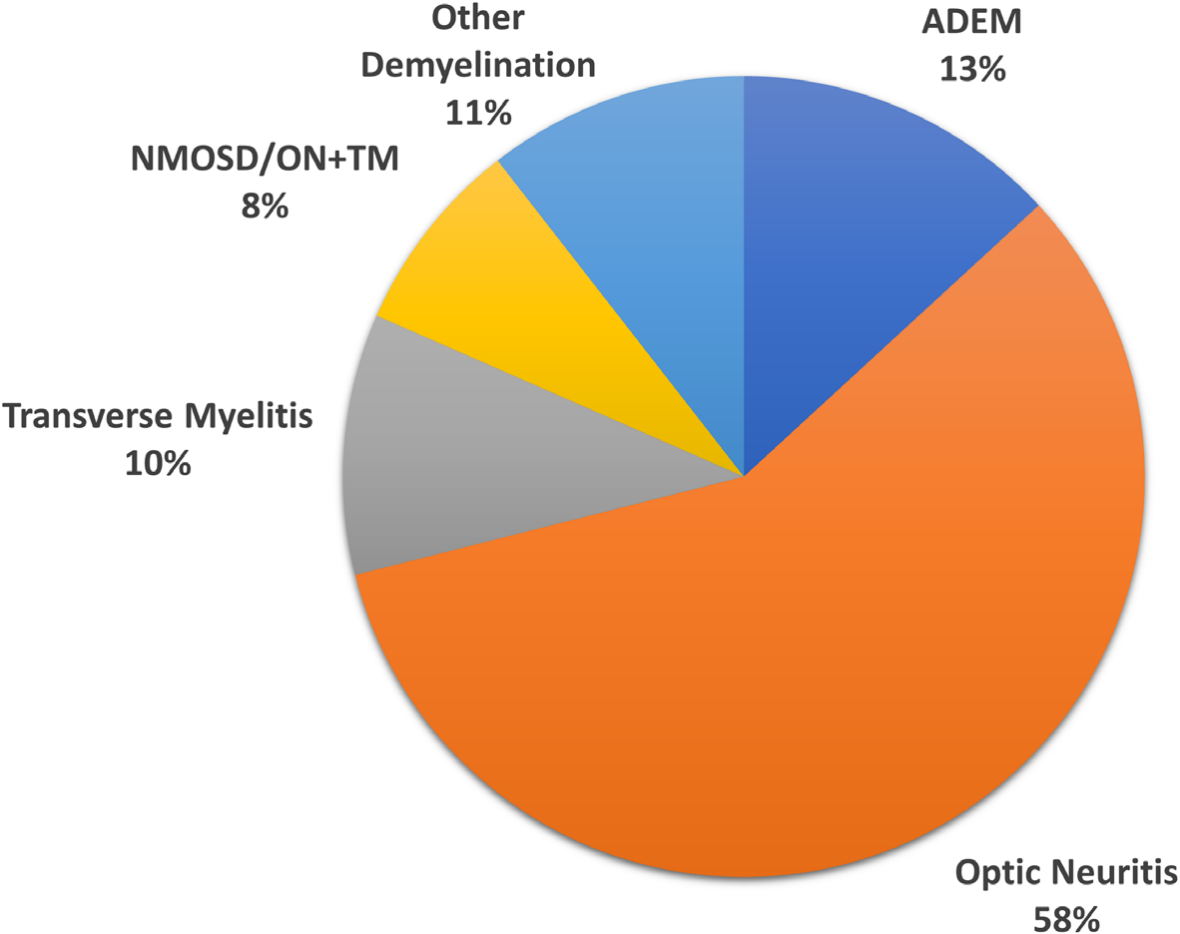
Initial clinical phenotype of first attack in pediatric MOGAD according to large inception cohorts for patients age ≥ 11 years (based upon^12^, ^15^).

The clinical phenotypes at first MOGAD presentation are summarized in Fig. 2 based on six large incident cohort studies.^11^, ^12^, ^13^, ^14^, ^15^, ^16^ Assessing these six studies individually, the percentage of children presenting with ADEM ranged from 38% to 66%, ON from 17% to 40%, and TM from 1% to 14%. The initial clinical phenotypes stratified by patients less than and greater than 11 years old are summarized in Figs. 3 and 4 according to two studies.^12^, ^15^ There was a clear effect of age on initial presentation when comparing Figs. 3 and 4, with patients older than 11 years old more than twice as likely to present with ON compared with patients younger than 11 years (58% versus 24%, respectively). The effect of age was stronger at even younger ages wherein 81% of children less than five years old presented with ADEM.^12^ Two studies assessed clinical phenotypes by sex and found no differences between males and females.^11^, ^12^

This general distribution of MOGAD phenotypes was also observed when assessing all identified studies (Table 1). Overall, 39 identified articles (including the six aforementioned incident cohort studies) reported the phenotype at initial presentation for 1,686 pediatric MOGAD patients.^4^, ^5^, ^6^, ^8^, ^11^, ^12^, ^13^, ^14^, ^15^, ^16^, ^17^, ^18^, ^19^, ^20^, ^21^, ^22^, ^23^, ^24^, ^25^, ^26^, ^27^, ^28^, ^29^, ^30^, ^31^, ^32^, ^33^, ^34^, ^35^, ^36^, ^37^, ^38^, ^39^, ^40^, ^41^, ^42^, ^43^, ^44^, ^45^ Most of these studies were limited by the potential for selection bias in patient identification (e.g., patients with stored serum available for retrospective MOG‐Ab testing, relapsing disease course, etc.). Nevertheless, when all patients from these articles were compiled, the results were highly similar to the six large inception cohorts: 43% ADEM, 31% ON, 10% TM, and 5% NMOSD/ON+TM. All articles that reported clinical phenotypes stratified by age confirmed that younger children are more likely to present with ADEM compared with older children and that older children are more likely to present with ON.

**Table 1:** Reported distribution of MOGAD attack phenotypes at disease presentation and during subsequent follow‐up according to all identified pediatric studies.

MOGAD phenotype	Initial presentation	Subsequent follow‐up^a^
	39 studies	35 studies
	1686 patients	1126 patients
ADEM or MDEM	43%	36%
ON	31%	19%
TM	10%	4%
NMOSD/ON+TM	5%	19%
ADEM+ON	–	6%
Other	11%	16%

ADEM, acute disseminated encephalomyelitis; MDEM, multiphasic disseminated encephalomyelitis; MOGAD, myelin oligodendrocyte glycoprotein antibody‐associated disease; MS, multiple sclerosis; NMOSD, neuromyelitis optica spectrum disorder; ON, optic neuritis; TM, transverse myelitis.

^a^ Includes patients with monophasic course.

### Relapsing course

The proportion of pediatric MOGAD patients with a relapsing course is approximately 23% (Table 2), based upon the six most informative studies reporting on 403 patients.^11^, ^12^, ^13^, ^14^, ^15^, ^16^ Four of the six studies reported results that clustered around the overall mean: 17%, 20%, 24%, and 28%.^11^, ^12^, ^15^, ^16^ In contrast, one study reported a substantially higher proportion of relapsing course (38%), but patients that were lost to follow‐up prior to two years of disease duration were excluded from the study (*N* = 40), and patients with a monophasic course may be more likely to be lost to follow‐up compared with patients with a relapsing course.^13^ One study reported a lower proportion of relapsing patients (11%), but the duration of follow‐up in this study was short (median approximately 12 months) and may not have been sufficient for disease relapses to occur.^14^ Consistent with the studies listed in Table 2, an additional study reported that the four‐year risk of relapse among patients aged <12 was 29% [95% confidence interval 16%–50%] and 24% [11%–48%] for patients aged 12 to 18 years.^8^

**Table 2:** Proportion of pediatric patients with MOGAD relapse according to large inception cohorts.

Study	Total *N*	Number with relapse	% with relapse	Duration of follow‐up (months)	Patient age at onset (years)
Armangue^11^	100	17	17%	Minimum 6	Median 6.2 (IQR 3.7, 10.0)
				Median 42 (IQR 22, 67)	
				Range 8–197	
Baumann^12^	69	19	28%	Minimum 12	Range 1–18
				Median 24; Mean 33	
				Range 12–84	
Hennes^13^	65	25	38%	Fixed at 24 for all patients	Median 6
					Range 0.5–17
Pedreno^14^	53	6	11%	Median 12^a^	Median 5^a^
					Range 0.1–18
				Range 0.3–143	
Waters^15^	82	16	20%	Minimum 6	Median 7.3 (IQR 4.9, 10.6)
				Median 82 (IQR 57, 106)	
Zhang^16^	34	8	24%	Minimum 12	Median 6.3
				Median 34.5	Range 1.6–14.2
				Range 14–63	
Total	403	91	23%		

IQR, interquartile range; MOGAD, myelin oligodendrocyte glycoprotein antibody‐associated disease; *N*, number of patients.

^a^ Approximated from published data.

The time to first MOGAD relapse is often within one year and typically within three years (Table 3). In the identified studies, there was relative clustering of the reported elapsed time to first relapse. Thirteen of 15 (87%) studies reported a median between four and 13 months. Most studies (5/7) reported that 75% of the first relapses occurred within three years, but this is partially dependent on the duration of follow‐up. The risk of first relapse may extend for a long period of time, as five of the identified studies overall reported observing initial relapses occurring more than 10 years after the first demyelinating event.^5^, ^10^, ^20^, ^27^, ^46^, ^47^

**Table 3:** Elapsed time to first relapse and number and frequency of relapses in pediatric MOGAD.

Article	Median elapsed months to first relapse (IQR)	Proportion with relapse within 1–2 Years	Distribution of number of relapses	Annualized relapse rate
Armangue^11^	NR	NR	Among patients with ≥1 relapse:	NR
			17/34 = 50% with 1	
			6/34 = 18% with 2	
			6/34 = 18% with 3	
			1/34 = 3% with 4	
			4/34 = 12% with ≥5	
Waters^15^	11.0 (3.7, 19.2)	NR	NR	Median 0.50 (IQR 0.50, 0.75)
Zhang^16^	13 (NR)	NR	Among patients with ≥1 relapse: Mean 1.25	NR
Dale^48^	NR	NR	NR	Median 0.56
Yoo^17^	4.2 (NR)	14/18 = 78% ≤ 1 year 16/18 = 89% ≤ 2 years	Among patients with ≥1 relapse: 15/18 = 83% with ≤2	NR
Cobo‐Calvo^6^	NR	38/52 = 73% ≤ 2 years	NR	Mean 0.23 (SD 0.35)
de Mol^5^	10.5 (NR)	NR	NR	NR
Deiva^20^	9 (5, 35)	NR	NR	Mean 0.36 (SD 0.82)
Hacohen^49^	4.5 (3.1, 8.1)	NR	NR	Median 1
Hacohen^4^	6.0 (NR)	NR	Among patients with ≥1 relapse: Median 2 (IQR 1, 4)	NR
Jarius^46^	63 (NR)	NR	NR	NR
Ramanathan^23^	NR	NR	Among patients with ≥1 relapse: Mean 2.4 Median 2 (Range 1–7)	NR
Senanayake^24^	NR	NR	Among patients with ≥1 relapse: Median 1 (Range 1–8)	NR
Chen^34^	NR	NR	Among patients receiving chronic immunotherapy: Median 5 (Range 1–11)	Mean 1.3 (Range 0–9.7)
Chen^25^	8 (NR)	NR	Among all patients: Median 1 (Range 0–4)	Median 0.88 (IQR 0.67, 2.53)
Cobo‐Calvo^26^	5.2 (NR)	NR	NR	NR
Fernandez‐Carbonell^50^	NR	NR	NR	Mean 0.42
Hacohen^35^	9.5 (3.75, 24)	NR	NR	NR
Konuskan^29^	NR	NR	Among all patients: Median 1 (Range 0–9) Mean 1.6 (SD 2.11)	Median 0.4 (Range 0–1.5) Mean 0.39 (SD 0.4)
Mao^31^	10 (NR)	NR	NR	NR
Zhou^37^	NR	NR	Among patients with ≥1 relapse: Median 2 (Range 1–7)	Median 1.29 (Range 0.56–4.0)
Ferilli^38^	NR	2/2 = 100% ≤ 2 years	Among patients with ≥1 relapse: Median 2	NR
Lui^51^	7.3 (NR)	NR	NR	Median 0.43
Azumagawa^58^	NR	NR	Among patients with ≥1 relapse: Mean 2.2	NR
Epstein^39^	8.9 (2.0, 36)	NR	NR	Median 1.11 (IQR 0.33, 1.36)
Li^42^	NR	NR	NR	Median 1.2 (Range 0–4)
Netravathi^43^	NR	NR	Among all patients: Mean 3.6 (SD 2.9)	NR
Rempe^47^	41	2/4 = 50% ≤ 1 year	NR	NR
Satukijchai^8^	NR	NR	NR	Mean 0.56

IQR, interquartile range; MOGAD, myelin oligodendrocyte glycoprotein antibody‐associated disease; NR, not reported; SD, standard deviation.

The rate of MOGAD relapse is often fewer than one event per year, and many patients will have only one or two relapses in total following the initial presentation. There was variation in the reported number of total relapses and the annualized relapse rate (ARR). Three of the larger studies reported that patients had an approximate median of two relapse events in total.^4^, ^11^, ^23^ Many of the identified studies reported a mean or median ARR between 0.4 and 1.1.^8^, ^15^, ^25^, ^29^, ^39^, ^48^, ^49^, ^50^, ^51^

### Subsequent attack phenotypes

**Figure 5:**
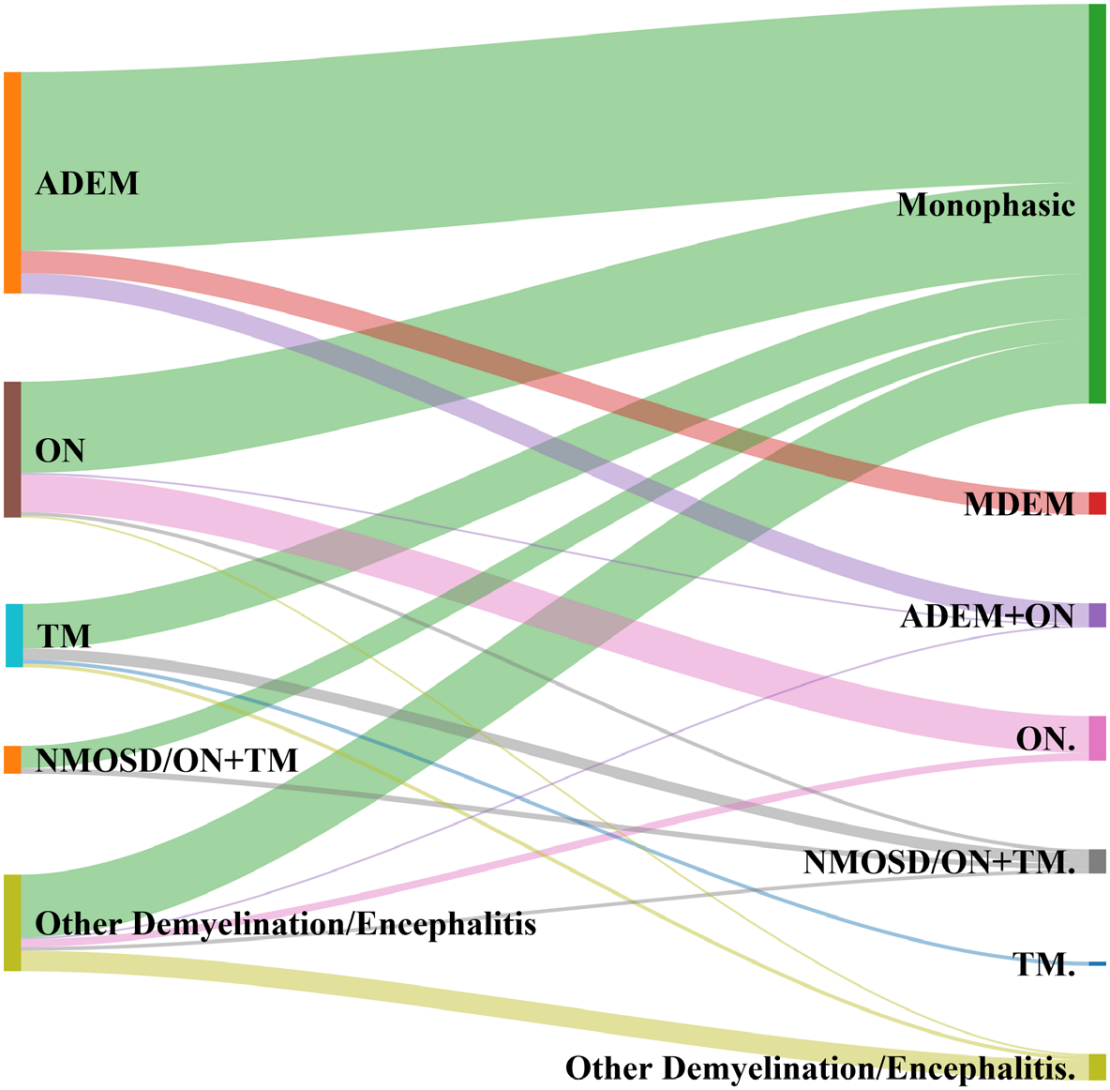
MOGAD initial phenotype and subsequent phenotype at most recent data collection. The initial phenotypes are shown on the left, and the most recent phenotypes are shown on the right. All patients with only one episode of MOGAD are categorized as “monophasic” for the most recent phenotype. Lines connecting the initial and most recent phenotypes represent individual patients' disease courses. The width of the lines is proportional to the number of patients observed with each disease course (based upon^11^, ^12^, ^16^, ^45^).

The clinical manifestations over the course of MOGAD are variable and may exhibit complex patterns. A diagram of MOGAD initial and subsequent phenotypes is shown in Fig. 5 based upon four large inception cohorts that collectively reported both the initial phenotype and the phenotype at the most recent data collection for 293 individual patients.^11^, ^12^, ^16^, ^45^ ON was present during the disease course (either in isolation or in combination with other manifestations) in approximately 64% of patients with at least one relapse. Approximately 18% of patients with a relapsing course had clinical phenotypes that did not fit classification criteria for any typical conditions (e.g., other demyelination, other encephalitis).

This general pattern of MOGAD phenotypes over time was also observed when assessing all identified studies (Table 1). Overall, 35 identified studies^4^, ^5^, ^8^, ^11^, ^12^, ^13^, ^14^, ^16^, ^19^, ^23^, ^24^, ^26^, ^27^, ^28^, ^29^, ^30^, ^31^, ^32^, ^35^, ^37^, ^38^, ^43^, ^49^, ^50^, ^52^, ^53^, ^54^, ^55^, ^56^, ^57^, ^58^, ^59^, ^60^, ^61^ reported MOGAD phenotypes during subsequent follow‐up for 1,126 pediatric patients. Studies showed substantial heterogeneity regarding patient identification/inclusion (e.g., restricted to patients with relapsing course or who received treatment with chronic immunosuppression) and phenotype assessment timing (e.g., at first disease relapse, at most recent follow‐up). Nevertheless, the overall distribution of MOGAD phenotypes during follow‐up (Table 1) was similar to the more systematic data from inception cohorts (Fig. 5). The proportions of patients with isolated ADEM/multiphasic disseminated encephalomyelitis (MDEM) or TM decreased during follow‐up and the proportion of patients with ON as a disease manifestation (including NMOSD and ADEM+ON) increased over time.

### Disease severity, disability and outcome measures

Many patients with MOGAD experience significant disability at the time of the acute presentation. Disease severity at presentation (or in some instances MOGAD relapse) was reported by 23 identified articles. The reported median EDSS scores ranged from 1.5^33^ to 5^16^, ^23^, ^62^ among the 14 studies reporting EDSS. One study reported EDSS scores stratified by phenotype and showed that children with TM were likely to have greater scores.^6^ All three studies reporting the mRS showed median scores of 3 to 4.^63^, ^64^, ^65^

The initial disability frequently subsequently improves in pediatric MOGAD. Disease severity after MOGAD onset was reported by 46 identified articles. The median subsequent EDSS scores were ≤1 in 25 of 28 (89%) studies. One study reported mRS of 0 in 83% of patients, with a range of 0–4.^65^ Three studies reported a median mRS of 1,^63^, ^66^ including one study that reported mRS of 1 for all 10 patients.^64^ Two studies reported mRS of ≥2 in 15%^11^ and 60% of patients.^30^

Visual acuity was reported only when patients had ON. Nine of 10 studies reporting visual acuity showed substantial visual impairment. Using the decimal scale, three studies each reported visual acuity of ≤0.2 occurring in 87%^6^ to 100%^16^, ^26^ of patients, and ≤0.1 occurring in 53%,^67^ 80%,^68^ and 82%^69^ of patients. One study reported a median visual acuity of 0.06.^62^ Conversely, one study of 10 patients reported a median visual acuity of 1.0 (interquartile range (IQR) 0.2, 1.0) using the decimal scale.^70^ Two studies reported the median logarithm of the minimal angle of resolution (logMAR) to be 1.7.^71^, ^72^

Studies reporting visual acuity consistently showed overall good to excellent visual outcomes following initial presentation in the majority of patients. Using the decimal scale, visual acuity of ≤0.2 was reported by three studies in 0%^6^, ^14^ to 20% of patients,^16^ and <0.5 by two studies in 2%^69^ and 6%^68^ of patients. Three studies reported visual acuity of ≥0.7 in 85%^26^; >0.8 in 89%^67^; and ≥1.0 in 89%.^31^ Two studies reported median visual acuity of 1.0, one with an IQR of 1.0, 1.0^70^ and one with a range of 0.8–1.0.^62^ The logMAR was reported by three studies: one with median 0.0 (IQR 0.0, 1.0),^72^ one with median 0.1,^71^ and one with logMAR 0.0 in 73% of patients.^73^

### Therapeutic utilization

The use of glucocorticoids and other immunosuppressive or immunomodulatory treatments was highly variable across identified studies and has been the topic of a recent review,^9^ but adequately controlled studies to quantitatively assess the effects of treatment on outcomes were not identified. In addition, the order and timing of administration of immunotherapy was heterogeneous, making it challenging to ascertain the impact of particular interventions on patient outcomes between studies.

### Risk factors for relapsing course

The published literature to date regarding risk factors for relapsing disease course is inconclusive. The persistence of MOG‐Ab was assessed as a potential risk factor for relapse by six published studies.^6^, ^10^, ^11^, ^15^, ^21^, ^74^ Three studies reported a strong association between persistent MOG‐Ab and relapse.^6^, ^10^, ^11^ However, this association was not observed by one study,^21^ and yet another study^15^ reported 5 of 16 (31%) of patients with relapse converted to MOG‐Ab negative status (including four patients with ≥1 relapse after converting to MOG‐Ab negative status). Additionally, because persistence of MOG‐Ab cannot be ascertained at the time of diagnosis and can only be observed over time, it cannot be used to guide decisions about initiation of maintenance therapy following acute treatment of the first demyelinating event.

The initial clinical phenotype was assessed as a risk factor for relapse by four studies.^11^, ^16^, ^17^, ^31^ Only one study^17^ reported a positive association between encephalopathy and a monophasic course, while the other three studies^11^, ^16^, ^31^ did not report any association between initial clinical phenotype and relapse.

No associations were found between relapsing course and age,^16^, ^31^ sex,^16^, ^31^ EDSS at onset,^16^ initial MOG‐Ab titer,^16^, ^75^ presence of IgA or IgM MOG‐Ab,^14^ cerebral spinal fluid pleocytosis or increased protein concentration,^16^ or location of MRI lesions.^16^, ^31^ No associations between relapsing course and various treatment characteristics were found, including acute treatment received at onset,^11^ duration of glucocorticoid treatment at onset,^16^ elapsed time to immunotherapy initiation,^16^ and response to initial therapy.^31^

## Discussion

Children with MOGAD have unique demographic and clinical features that both differentiate them from adult populations and highlight the challenging aspects of diagnosis and prognosis. Data from inception cohorts indicate that nearly one‐half of children with MOGAD present with ADEM and approximately one‐quarter with ON. Among children 11 years and older, ON is more frequent and ADEM is less frequent, more closely mirroring clinical phenotypes observed in adults.^6^, ^76^ Although the methods of many of the identified studies contained selection bias, when all reports were pooled, the resulting proportions of pediatric MOGAD phenotypes was similar to the inception cohorts. The broad range of phenotypes observed in pediatric MOGAD is important to clinicians because nearly all demyelinating phenotypes have been linked to the presence of MOG‐Ab often requiring nuanced monitoring and therapeutic approaches.^4^

Evaluation of the risk of relapse in children with MOGAD is highly relevant as it can inform the need for clinical and radiographic screening. Following initial presentation of MOGAD, approximately one‐quarter of patients will have a subsequent relapse, although this estimate may be higher with longer durations of follow‐up, as multiple studies identified longer quiescent periods than are typically observed in other pediatric demyelinating diseases.^5^, ^10^, ^20^, ^27^, ^46^, ^47^ Nevertheless, it appears the majority of first relapses occur within the first two years of diagnosis with most patients having one or fewer relapses per year. Importantly, when relapses occur the clinical phenotype may change, with the most common variation being the development of ON at subsequent attacks. Variable clinical presentations at relapse are highly relevant in children with MOGAD as this is rarely observed in other pediatric demyelinating disorders such as NMOSD and non‐MOG‐Ab associated ON and TM. Although higher rates of relapsing MOGAD have been reported in children, interpretation of these data are challenging given the risk of bias associated with convenience cohorts and severity bias in testing. A significant finding from this study is that relapses have been reported in both children who remain seropositive and those who do not, albeit the latter group having significantly lower rates of recurrent disease.^15^ The studies did not discriminate risk of relapse by specific age groupings (e.g., <6 years, 6–11 years, and greater than 11 years) which anecdotally has produced a spectrum of greater relapsing risk as age increases. Further systematic studies on pediatric relapse risk are needed, as such data are critical to the treating neurologist to assist with screening and prognostic recommendations provided to patients.

The lack of reliable predictors of relapsing disease aside from age is an additional knowledge gap in MOGAD with multiple typical risk factors for demyelinating disease being inconclusive.^6^, ^10^, ^11^, ^14^, ^15^, ^16^, ^17^, ^21^, ^31^, ^74^, ^75^ Other potential risk factors for relapsing course have been suggested by observations in clinical practice, including older patient age, lower spinal cord involvement, and race/ethnicity, but these have not yet been substantiated in published studies. Accurately predicting a patient's likelihood of relapse is important for screening and potential prophylactic management for children with MOGAD.

Most studies report a favorable clinical outcome in children with MOGAD, but a minority of children may experience a more severe course causing neurologic sequelae. However, current measures of disease activity, severity, and disability appear insufficient and nonspecific for MOGAD evaluation. Assessment of disease activity and disability in MOGAD is a particular knowledge gap and therefore a high need area further research. For example, EDSS, developed for use in adults with MS, was the most commonly reported measure, but the score is heavily weighted toward motor function relative to cognitive function, and cognitive function may be an important manifestation of ADEM, especially long‐term.^20^ Only one of the 14 studies reporting visual acuity explicitly stated that low‐contrast testing was performed^73^; therefore, milder visual abnormalities may not be identified. Neuropsychological outcomes are important but testing is often difficult to access with only one article reporting detailed outcomes on this.^61^ Reliable assessment of cognition remains a high research priority due to the potential impact on quality of life for children in both childhood and adulthood. In addition to better measures of MOGAD, longer follow‐up of patients with a relapsing course is needed to assess for possible cumulative damage.

This study is not without limitations. First, this review included both retrospective and prospective studies on MOGAD in children which utilized different parameters with regards to diagnosis, age range, disease severity and disability assessment, and time from of monitoring for relapse. Expectedly, this creates difficulties when comparing studies although standardized data extraction was utilized to mitigate this impact. The authors attempted to be as broad as possible with inclusion criteria in order to capture the largest amount of data for review. Thus, data reported in this study should be viewed in the context of trends observed in children with MOGAD as opposed to definitive statements on clinical phenotypes and natural history of the disease. Similarly, given the heterogeneity of studies included, definitions of phenotypes differed between studies. For instance, among the studies contributing data to Fig. 5, patients were labeled as having NMOSD using Wingerchuck 2015 criteria^77^ in two studies,^11^, ^16^ the International Pediatric Multiple Sclerosis Study Group criteria^78^ in another study,^12^ and nonspecific “ON + TM” in another.^45^ This was also relevant when terminology such as “other encephalitis” which referred to encephalitis not conforming to ADEM criteria. In addition, many of the identified articles were limited by selection bias which is anticipated given the limited data available on MOGAD in children. Multiple studies were based on convenience cohorts of patients who presented to academic specialty centers and were thought to be most reflective of moderate and severe presentations of illness as well as having higher access to appropriate MOG‐Ab testing. Furthermore, with a few exceptions, studies did not necessarily identify patients with MOGAD at initial presentation. This limitation should continue to resolve with increased recognition of MOGAD and availability of MOG‐Ab testing as part of the initial clinical evaluation of demyelination. In earlier publications (specifically prior to commercialized MOG‐Ab testing) treating physicians may have more likely ordered antibody testing if specific characteristics (e.g., higher disease severity, slower resolution of disease manifestations) were present, creating a selection bias and increased likelihood of relapsing disease capture. Further, several studies included patients who did not have MOG‐Ab testing performed at the time of the first event but rather following relapses of disease (Fig. 1). Moreover, monophasic patients without MOG‐Ab testing at the first event are unlikely to ever be tested. As a result, many studies contained an over‐representation of patients with relapsing MOGAD. As noted by the EU Pediatric MOG Consortium (1): “The frequency of relapses among all paediatric MOG‐Ab‐positive patients ranges widely between studies, because of e.g., differences in follow‐up time, study design (retro‐ vs. prospective) and patient selection (nationwide coverage vs. secondary academic centres).” Another source of variation is the timing of MOG‐Ab testing. Approximately two‐thirds of the identified studies not included in Table 2 (i.e., not inception cohorts) reported the proportion of pediatric MOGAD patients with a relapsing course to be greater than 30%; however, this is likely a result of selection bias. For example, in one study the proportion of pediatric and adult MOGAD patients with a relapsing course was 50% among the entire identified study cohort (i.e., a convenience sample) but only 25% when restricted to patients with an incident diagnosis who were identified prospectively (42). With regards to relapses, it is important to note that number and frequency of relapses reported is highly dependent upon the duration of the follow‐up and the effectiveness of any chronic treatments received, and these factors varied substantially across studies making definitive interpretation difficult.

In conclusion, MOGAD in children represents a highly heterogenous clinical spectrum of disease with approximately one‐quarter of patients developing a relapsing course. This study highlights that in children with relapsing MOGAD, clinical phenotypes of individual patients may change over time and that there were no specific predictors of development of a relapsing course across multiple studies. Further prospective, multi‐center longitudinal data collection on children with MOGAD is desperately needed. Studies on effective therapies, relapse prediction, and cognitive impact remain high research priorities.

## Author Contributions

TB, SRKH, and FT conceived and designed the study. TB and SRKH acquired the data. All authors interpreted the data. JDS and TB drafted the initial manuscript. All authors critically reviewed the manuscript and approved its final form.

## Conflict of Interest Statement

This study was funded by UCB Pharma. Medical writing support was provided by Epi Excellence LLC (Garnet Valley, PA, USA) and funded by UCB Pharma, in accordance with Good Publication Practice (GPP3) guidelines (ismpp.org/gpp3). JDS receives compensation from UCB on the topic of MOGAD. TB has received consulting fees from UCB for participation in a Data Safety Monitoring Board, unrelated to this work. CH has received honoraria from Novartis and Sanofi and consulting fees from UCB Pharma, Novartis, Biogen, Roche, and VeilaBio. SRKH was an employee of UCB Pharma at the time of the study. FT is an employee and shareholder of UCB Pharma. TC has received compensation for consulting from Banner Life Sciences, Biogen, Bristol‐Myers Squibb, Janssen, Novartis Pharmaceuticals, Roche Genentech, Sanofi Genzyme, and UCB Pharma; and research support from the National Institutes of Health, National MS Society, US Department of Defense, Sumaira Foundation, Brainstorm Cell Therapeutics, Bristol‐Myers Squibb, EMD Serono, I‐Mab Biopharma, Mallinckrodt ARD, Novartis Pharmaceuticals, Octave Bioscience, Roche Genentech, Sanofi Genzyme, and Tiziana Life Sciences.

## Data Availability

Data from non‐interventional studies is outside of UCB's data sharing policy and is unavailable for sharing.
